# H19 and IGF-2 allele-specific expression in hepatoblastoma

**DOI:** 10.1054/bjoc.1999.0992

**Published:** 2000-02

**Authors:** J A Ross, G A Radloff, S M Davies

**Affiliations:** Department of Pediatrics and Clinical Research Division of Hematology-Oncology and Bone Marrow Transplantation, Box 422, 420 Delaware St SE, Minneapolis, MN 55455, USA; University of Minnesota Cancer Center, Minneapolis, MN, USA

**Keywords:** IGF-2, H19, hepatoblastoma, genomic imprinting

## Abstract

Patterns of allele-specific expression of H19 and insulin-like growth factor-2 (IGF-2) were examined in tissue obtained from 30 children diagnosed with hepatoblastoma. All informative tumours demonstrated monoallelic expression of H19. In contrast, variable patterns of allele-specific expression of IGF-2 were seen in tumours from children of different ages.© 2000 Cancer Research Campaign


British Journal of Cancer (2000) **82**(4), 753–756
© 2000 Cancer Research Campaign
DOI: 10.1054/ bjoc.1999.0992, available online at http://www.idealibrary.com on IDEAL®

### H19 and IGF-2 allele-specific expression in hepatoblastoma

**JA Ross[1,3], GA Radloff[2,3] and SM Davies[2,3]**

Department of Pediatrics, [1]Division of Pediatric Epidemiology and Clinical Research, and [3]Division of Pediatric Hematology-Oncology and Bone Marrow Transplantation, Box 422, 420 Delaware St SE, Minneapolis, MN 55455, USA; [3]University of Minnesota Cancer Center, Minneapolis, MN, USA

**Summary** Patterns of allele-specific expression of H19 and insulin-like growth factor-2 (IGF-2) were examined in tissue obtained from 30 children diagnosed with hepatoblastoma. All informative tumours demonstrated monoallelic expression of H19. In contrast, variable patterns of allele-specific expression of IGF-2 were seen in tumours from children of different ages. © 2000 Cancer Research Campaign

**Keywords:** IGF-2; H19; hepatoblastoma; genomic imprinting

Genomic imprinting is the differential expression of a gene depending on the parent of origin. H19 and insulin-like growth factor-2 (IGF-2) are imprinted genes that map to a 90-kilobase (kb) region on chromosome 11p15 (Zemel et al, 1992). In the majority of human tissues, only the maternal allele of the H19 gene is expressed and only the paternal allele of the IGF-2 gene is expressed. In adult human liver and fetal choroid plexus and leptomeninges, however, IGF-2 is biallelically expressed (Ohlsson et al, 1993; Ekstrom et al, 1995).

In human liver, IGF-2 is monoallelically expressed at birth, with a switch to biallelic expression some time during the first year of post-natal life (Ohlsson et al, 1993; Davies, 1994; Li et al, 1996). IGF-2 expression is regulated by four promoters, P1–P4, which are expressed, and hence imprinted, differently. P1 is biallelically expressed and is active in adult human liver. In fetal and early post-natal liver, however, the pattern of P1 expression is complex. Although it has been demonstrated that the P1 promoter directs expression from both parental alleles in prenatal as well as post-natal liver specimens (Ekstrom et al, 1995), some studies suggest that the P1 promoter is not used in early fetal life (Li et al, 1996, 1998), while others have detected P1-derived transcripts in fetal and newborn liver tissue (Taniguchi et al, 1995; Vu and Hoffman, 1996). In contrast, promoters P2–P4 are expressed monoallelically and are used in fetal and infant tissues, but in older infants (> 18 months) and adults imprinting of P2–P4 exhibits plasticity. Both monoallelic and biallelic expression have been described, as have instances of monoallelic expression from opposite parental alleles. Switching from monoallelic to biallelic IGF-2 expression in normal human liver in the first year of life involves increased use of P1 and the development of plasticity in P2–P4. In contrast, H19 is imprinted in normal human liver throughout life (Ekstrom et al, 1995). The precise mechanism by which genes and their regulatory elements are imprinted is currently unknown, although available data suggest that methylation of DNA may determine the imprint (Hu, 1996).

*Received 18 March 1999*
*Revised 2 September 1999*
*Accepted 23 September 1999*

*Correspondence to:* JA Ross

Biallelic expression of IGF-2 and H19 has been demonstrated in several paediatric and adult malignancies, including Wilms' tumour, Ewing sarcoma, embryonal rhabdomyosarcoma, germ cell tumours, lung cancer, oesophageal cancer, glioma and renal cell carcinoma (Glassman et al, 1996; Uyeno et al, 1996; Nonomura et al, 1997; Ross et al, 1999). In contrast, for hepatoblastoma, an embryonal liver tumour that usually occurs in the first 5 years of life, monoallelic expression of IGF-2 has generally been reported, with biallelic expression observed in only a minority of cases (Davies, 1993; Li et al, 1995; Rainier et al, 1995). In this study, we examined expression of IGF-2 and H19 in informative tumour specimens and normal matched tissue (if available) from children of different ages diagnosed with hepatoblastoma.

### MATERIALS AND METHODS

### Tumour samples

Hepatoblastoma tissue (and normal matched tissue, if available), frozen at –70°C, was obtained from the Cooperative Human Tissue Network (CHTN; Columbus, OH, USA). Tissue underwent institutional and central pathological review to ensure uniformity of diagnosis. Normal liver tissue from a 14-year-old traffic accident victim was obtained from the Liver Tissue Procurement and Distribution System (NIH Grant N01-DK-6-2274).

### Allele-specific gene expression

Genomic DNA (gDNA) was extracted using standard techniques, and samples were screened for heterozygosity for a known *Apa*I polymorphism within exon 9 of IGF-2 as previously described (Ogawa et al, 1993). If heterozygous (and hence informative), reverse transcription polymerase chain reaction (RT-PCR) was performed as described by Nonomura et al (1997). PCR products were digested with *Apa*I (yielding either a 236-bp fragment or a 173- and 63-bp fragment), electrophoresed through a 1.5% agarose gel, and visualized with ethidium bromide. To monitor for gDNA contamination of the cDNA preparation, RT-PCR was performed both with and without reverse transcriptase; no amplification was observed from samples without reverse transcriptase (Figure 1A).

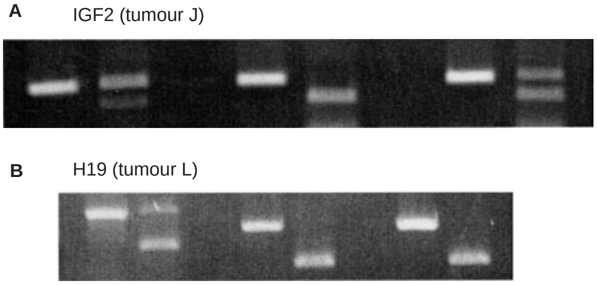

**Figure 1** (**A**) IGF-2, tumour J. Each panel contains nine lanes: Lane 1 contains primer-amplified gDNA, Lane 2 contains primer amplified gDNA digested with *Apa*I to demonstrate heterozygosity, Lane 3 is a control to monitor for gDNA contamination of the tumour cDNA product (cDNA without reverse transcriptase), Lane 4 contains primer-amplified cDNA product, Lane 5 contains primer-amplified cDNA tumour product digested with *Apa*I, Lane 6 is a control to monitor for gDNA contamination of cDNA from normal liver adjacent to the tumour, Lane 7 contains primer-amplified cDNA from normal tissue, and Lane 8 contains primer-amplified cDNA from normal tissue digested with *Apa*I (**B**) H19, tumour L. Each panel contains nine lanes: Lane 1 contains primer-amplified gDNA, Lane 2 contains primer amplified gDNA digested with *Rsa*I to demonstrate heterozygosity, Lane 3 is a control to monitor for gDNA contamination of the tumour cDNA product (cDNA without reverse transcriptase), Lane 4 contains primer-amplified cDNA product, Lane 5 contains primer-amplified cDNA tumour product digested with the *Rsa*I, Lane 6 is a control to monitor for gDNA contamination of cDNA from normal liver tissue adjacent to the tumour, Lane 7 contains primer-amplified cDNA from normal tissue, and Lane 8 contains primer-amplified cDNA from normal tissue digested with *Rsa*I

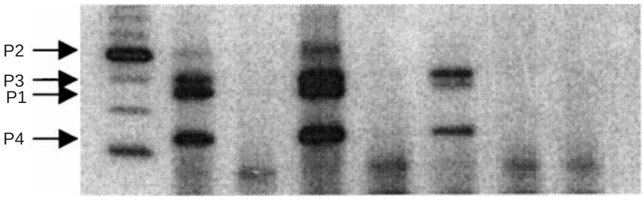

**Figure 2** IGF-2 promoter usage. Lane 1 contains a size marker, Lane 2 contains promoter-specific primer amplified cDNA product (P2 = 277 bp, P3 = 211 bp, P1 = 186 bp, P4 = 119 bp) of liver tissue obtained from a 14-year-old traffic accident victim, Lanes 3, 5 and 7 are controls to monitor for gDNA contamination of the cDNA product, Lane 4 contains promoter-specific primer amplified cDNA product from normal liver tissue adjacent to tumour M, Lane 6 contains promoter-specific primer amplified cDNA product from normal liver tissue adjacent to tumour G, Lane 8 is a PCR-negative control. (Although P2 appears faint in Lane 6, it is clearly apparent with longer exposure)

IGF-2 promoter usage was evaluated using the method of Hu et al (1996) (Figure 2). In brief, a multiplex PCR was performed in which cDNAs were amplified using four promoter-specific 5′-primers and a common 3′-primer end-labelled with [$\gamma$-$^{32}$P]ATP. Oligonucleotide primers used were: CAGTCCTGAGGTGAGCT-GCTGTGGC (hP1), ACCGGGCATTGCCCCCAGTCTCC (hP2), CGTCGCACATTCGGCCCCCGCGACT (hP3), TCCTCCTCCT-CCTGCCCCAGCG (hP4), CAGCAATGCAGCACGAGGCGA-AGCC (3′ primer).

Amplification of H19 was performed as described by Uyeno et al (1996). PCR products were digested with *Rsa*I (yielding either a 655-bp product or a 485- and 170-bp fragment). If heterozygous, cDNA was reverse transcribed and amplified in the same manner as gDNA producing 575- or 405-bp products after digestion with *Rsa*I. As the primers span the last intron of H19, gDNA contamination is excluded by the absence of a 655 bp product (Figure 1B).

For all experiments, PCR amplification and digests were repeated in duplicate to exclude the possibility of incomplete digestions yielding misleading results. In addition, all samples were digested in the presence of a positive control homozygous for the smaller fragment to ensure complete digestions. To monitor for potential cross-sample DNA contamination of PCR reactions, control amplifications were performed without DNA template and were shown to be negative for each experiment.

### RESULTS

Genomic DNA from 30 hepatoblastomas was amplified by PCR and digested with *Apa*I and *Rsa*I to identify the presence of previously described polymorphisms in IGF-2 and H19 respectively (Ogawa et al, 1993; Rainier et al, 1993). Thirteen (43%) tumours were informative (heterozygous) for H19, and 13 were informative for IGF-2. Six (20%) hepatoblastomas were heterozygous for both IGF-2 and H19. Four tumours had normal adjacent liver tissue available (Table 1). Demographic data on individuals with these informative tumours are provided in Table 1. The majority (80%) of patients were male and the median age at diagnosis was 1.8 years (range 1 month to 9 years).

For all tissue examined (including both malignant and normal adjacent liver tissue), H19 was monoallelically expressed. In contrast, variable patterns of allele-specific expression at IGF-2 were observed. The majority (10/13) of informative tumours demonstrated monoallelic expression of IGF-2. Tumours demonstrating biallelic expression of IGF-2 (E, I and T) were diagnosed in children aged 10 months, 18 months and 9 years respectively. In two cases (J and L; diagnosed at ages 18 months and 2 years) normal adjacent liver tissue showed biallelic expression of IGF-2, while expression was monoallelic in the tumour tissue. However, two others (G and M; diagnosed at ages 13 months and 2 years) showed monoallelic IGF-2 expression in tumour tissue and in adjacent normal liver tissue.

Examination of IGF-2 promoter usage in the adjacent normal liver tissue of G and M showed usage of all promoters, including P1 (Figure 2).

### DISCUSSION

This study represents one of the largest investigations of H19 and IGF-2 allele expression in hepatoblastoma. Allele-specific expression in hepatoblastoma is of particular interest since monoallelic expression of IGF2 is typically observed in normal fetal liver, with a change to biallelic expression occurring during the first year of life. This pattern is in direct contrast to that observed in most other normal tissues where monoallelic expression of IGF-2 continues throughout life. We examined allele-specific expression of IGF-2 and H19 in a wide age range of hepatoblastomas including children diagnosed in the first few months of life, as well as tumours diagnosed at 2 years of age or greater, where biallelic IGF-2 expression is expected in normal liver.

We found monoallelic expression of H19 was maintained in all tissue (malignant and normal) studied. Few previous studies have specifically examined H19 allele expression in hepatoblastoma. Rainier et al (1995) examined five hepatoblastomas and found that H19 was biallelically expressed in one tumour that demonstrated biallelic expression of IGF-2. In contrast, in a study of three tumours, Li et al (1995) reported that monoallelic expression was

*© 2000 Cancer Research Campaign*

**Table 1** Allelic usage of IGF2 and H19 in 20 informative hepatoblastoma tumours

| ID | Gender | Normal tissue | Age at Dx | IGF-2 informative | H19 informative | IGF-2 (malignant tissue) | H19 (malignant tissue) | IGF-2 (normal adjacent tissue) | H19 (normal adjacent tissue) |
|---|---|---|---|---|---|---|---|---|---|
| A | M | | 1 months | Yes | No | Monoallelic | | | |
| B | M | | 7 months | Yes | Yes | Monoallelic | Monoallelic | | |
| C | M | | 8 months | No | Yes | | Monoallelic | | |
| D | M | | 10 months | No | Yes | | Monoallelic | | |
| E | F | | 10 months | Yes | Yes | Biallelic | Monoallelic | | |
| F | M | | 12 months | Yes | Yes | Monoallelic | Monoallelic | | |
| G | M | Yes | 13 months | Yes | No | Monoallelic | | Monoallelic | |
| H | F | | 15 months | No | Yes | | Monoallelic | | |
| I | M | | 18 months | Yes | No | Biallelic | | | |
| J | M | Yes | 18 months | Yes | Yes | Monoallelic | Monoallelic | Biallelic | Monoallelic |
| K | F | | 2 years | Yes | No | Monoallelic | | | |
| L | F | Yes | 2 years | Yes | Yes | Monoallelic | Monoallelic | Biallelic | Monoallelic |
| M | M | Yes | 2 years | Yes | Yes | Monoallelic | Monoallelic | Monoallelic | Monoallelic |
| N | F | | 2 years | Yes | No | Monoallelic | | | |
| O | F | | 2 years | No | Yes | | Monoallelic | | |
| P | M | | 2.5 years | Yes | No | Monoallelic | | | |
| Q | M | | 2.5 years | No | Yes | | Monoallelic | | |
| R | M | | 3 years | No | Yes | | Monoallelic | | |
| S | M | | 4 years | No | Yes | | Monoallelic | | |
| T | M | | 9 years | Yes | No | Biallelic | | | |

maintained for H19 in a tumour that demonstrated biallelic expression of IGF-2. Given that all of our informative hepatoblastomas demonstrated monoallelic expression at H19, it is unlikely that alterations in allele-specific expression of H19 plays a major role in the development of hepatoblastoma.

In contrast to H19, variable patterns of allele-specific expression of IGF-2 were observed. The majority of tumours in our study demonstrated monoallelic expression of IGF-2, supporting observations reported in smaller studies (Davies, 1993; Li et al, 1995; Rainier et al, 1995). Biallelic expression of IGF-2 in malignant tissue at most sites is abnormal and is considered part of the malignant phenotype, potentially contributing to the progression of disease. In contrast, biallelic expression of IGF-2 is considered normal in post-natal human liver and fetal choroid plexus/leptomeninges (Ekstrom et al, 1995). Switching from the fetal pattern of monoallelic IGF-2 expression to biallelic expression in normal human liver development entails increased use of the biallelically expressed promoter P1, together with plasticity of imprinting of P2–P4 (Li et al, 1998). The precise age at which this typically occurs is controversial and has only been studied in a small number of cases. Some studies suggest that biallelic IGF-2 expression begins between 6 months and 1 year post-natal age (Ohlsson et al, 1993; Davies, 1994), whereas others observed biallelic IGF-2 expression in the liver tissue of a 2-month-old (Li et al, 1996). In the latter study, however, the tissue evaluated was adjacent normal liver from a child with hepatoblastoma, which may not be appropriate for determination of developmental patterns in healthy individuals.

In our study, the observation of monoallelic IGF-2 expression in hepatoblastoma tissue at ages when normal liver expresses IGF-2 biallelically (tumours J, K, L, M, N, P; all 18 months of age or older) may indicate a failure to follow the normal sequence of change in promoter usage (e.g. increased usage of P1). In such cases, it is possible that carcinogenesis is initiated at an earlier developmental time point than in cases in which normal progression to biallelic usage is seen. Moreover, the observation of monoallelic IGF-2 expression in normal liver adjacent to hepatoblastoma may indicate premalignant change in a larger area of the liver than that morphologically involved with tumour. Nevertheless, for the two cases (G and M; ages 13 months and 2 years) that showed monoallelic expression of IGF-2, all four promoters were utilized in the normal adjacent liver tissue. If the age at which promoter switching occurs is more variable than that reported to date, it is possible that the normal liver in these cases has simply not undergone the switch from monoallelic to biallelic IGF-2 expression and that the allele usage observed in both tumour and normal liver is appropriate, and not related to the malignant phenotype. Clearly, larger studies of normal liver tissue obtained in the first few years of life are needed to accurately evaluate the variability of timing of promoter silencing and switching of IGF-2.

In malignancies other than hepatoblastoma, allele-specific expression patterns for H19 and IGF-2 appear to vary, with coupling of expression frequently observed. For example, in testicular germ cell tumours (both paediatric and adult), as well as in Wilms' tumour, biallelic expression of both H19 and IGF-2 has been frequently observed (van Gurp et al, 1994; Nonomura et al, 1997, Ross et al, 1999). In childhood embryonal tumours in particular, biallelic expression of IGF-2 is thought to be related to elevated levels of IGF-2 production, and potentially to contribute to growth and progression of the tumour (Leisenring et al, 1994). An alternative interpretation of these data is that biallelic expression of IGF-2 represents non-specific gene dysregulation, perhaps due to genome-wide hypomethylation as part of the malignant phenotype as was shown in hepatocellular carcinoma (Takeda et al, 1996; Li et al, 1997). While our data regarding hepatoblastomas could represent non-specific dysregulation of gene expression, the continuation of monoallelic expression would argue against hypomethylation as a mechanism. Li et al (1995) suggest that for hepatoblastoma (unlike Wilms' tumour) the level of H19 expression is not a prerequisite for maintaining a monoallelic IGF-2 expression. Moreover, as in our study, they report monoallelic expression of H19 in a case of hepatoblastoma that showed biallelic expression of IGF2.

© 2000 Cancer Research Campaign 

In summary, we conclude that monoallelic expression of H19 is maintained in the vast majority of hepatoblastomas, suggesting that alterations of allele-specific expression of H19 are unlikely to play a major role in carcinogenesis at this site. Moreover, we report variable allele-specific expression patterns for IGF-2 with respect to age at diagnosis. To further understand the importance of IGF-2 allele-specific expression in the malignant phenotype, larger studies evaluating the age at which the switch from mono-allelic to biallelic IGF-2 expression occurs in normal liver are warranted.

### ACKNOWLEDGEMENTS

Supported by a grant from the Children's Cancer Research Fund and NIH Grant N01-DK-6-2274. Hepatoblastoma tissue and normal matched liver tissue was kindly provided by the Children's Cancer Group and the Pediatric Oncology Group through the Pediatric Division of the Cooperative Human Tissue Network funded by the National Cancer Institute. The authors wish to thank Peter Schmidt and Catherine Moen for technical assistance.

### REFERENCES

Davies SM (1993) Maintenance of imprinting at the IGF-2 locus in hepatoblastoma. *Cancer Res* **53**: 4781–4783

Davies SM (1994) Developmental regulation of genomic imprinting of the IGF-2 gene in human liver. *Cancer Res* **54**: 2560–2562

Ekstrom TJ, Cui H, Li X and Ohlsson R (1995) Promoter-specific IGF-2 imprinting status and its plasticity during human liver development. *Development* **121**: 309–316

Glassman ML, de Groot N and Hochberg A (1996) Relaxation of imprinting in carcinogenesis. *Cancer Genet Cytogenet* **89**: 69–73

Hu J-F, Vu TH and Hoffman AR (1996) Promoter-specific modulation of insulin-like growth factor II genomic imprinting by inhibitors of DNA methylation. *J Biol Chem* **30**: 18253–18262

Leisenring WM, Breslow NE, Evans IE, Beckwith JB, Coppes MJ and Grundy P (1994) Increased birthweights of National Wilms' Tumor Study patients suggest a growth factor excess. *Cancer Res* **54**: 4680–4683

Li X, Adam G, Cui H, Sandstedt B, Ohlsson R and Ekstrom TJ (1995) Expression, promoter usage, and parental imprinting status of insulin-like growth factor 2 (IGF-2) in human hepatoblastoma: uncoupling of IGF-2 and H19 imprinting. *Oncogene* **11**: 221–229

Li X, Cui H, Sandstedt B, Norlinder H, Larsson E and Ekstrom TJ (1996) Expression levels of the insulin-like growth factor-2 gene (IGF-2) in the human liver: developmental relationships of the four promoters. *J Endocrinol* **149**: 117–124

Li X, Non Z, Ekstrom C, Larsson E, Nordlinder H, Hofmann WJ, Trautwein C, Odenthal M, Dienes HP, Ekstrom TJ and Schirmacher P (1997) Disrupted IGF-2 promoter control by silencing of promoter P1 in human hepatocellular carcinoma. *Cancer Res* **57**: 2048–2054

Li X, Gray SG, Flam F, Pietsch T and Ekstrom TJ (1998) Developmental-dependent DNA methylation of the IGF-2 and H19 promoters is correlated to the promoter activities in human liver development. *Int J Dev Biol* **42**: 687–693

Nonomura N, Nishimura K, Miki T, Kanno N, Kojima Y, Yokoyama M and Okuyama A (1997) Loss of imprinting of the insulin-like growth factor 2 gene in renal cell carcinoma. *Cancer Res* **57**: 2575–2577

Ogawa O, Eccles MR, Szeto J, McNoe LA, Yun K, Maw MA, Smith PJ and Reeve AE (1993) Relaxation of insulin-like growth factor 2 gene imprinting implicated in Wilms' tumour. *Nature* **362**: 749–751

Ohlsson R, Nystrom A, Pfeifer-Ohlsson S, Tohonen V, Hedborg F, Schofield P, Flam F and Ekstrom TJ (1993) IGF-2 is parentally imprinted during human embryogenesis and in the Beckwith–Wiedemann syndrome. *Nat Genet* **4**: 94–97

Rainier S, Johnson LA, Dobry CJ, Ping AJ, Grundy PE and Feinberg AP (1993) Relaxation of imprinted genes in human cancer. *Nature* **362**: 747–749

Rainier S, Dobry CJ and Feinberg AP (1995) Loss of imprinting in hepatoblastoma. *Cancer Res* **55**: 1836–1838

Ross JA, Schmidt PT, Perentesis JP and Davies SM (1999) Genomic imprinting of IGF-2 and H19 in pediatric germ cell tumours. *Cancer* **85**: 1389–1394

Takeda S, Kondo M, Kumada T, Koshikawa T, Ueda R, Nishio M, Osada H, Suzuki H, Nagatake M, Washimi O, Takagi K, Takahashi T, Nakao A and Takahashi T (1996) Allelic-expression imbalance of the insulin-like growth factor 2 gene in hepatocellular carcinoma and underlying disease. *Oncogene* **12**: 1589–1592

Taniguchi T, Schofield AE, Scarlett JL, Morison IM, Sulllivan MJ and Reeve AE (1995) Altered specificity of IGF-2 promoter imprinting during fetal development and onset of Wilms' tumour. *Oncogene* **11**: 751–756

Uyeno S, Aoki Y, Nata M, Sagisaka K, Kayama T, Yoshimoto T and Ono T (1996) IGF-2 but not H19 shows loss of imprinting in human glioma. *Cancer Res* **56**: 5356–5359

van Gurp RJHLM, Oosterhuis JW, Kalscheuer V, Mariman ECM and Looijenga LHJ (1994) Biallelic expression of the H19 and IGF-2 genes in human testicular germ cell tumors. *J Natl Cancer Inst* **86**: 1070–1075

Vu TH and Hoffman AR (1996) Alterations in the promoter-specific imprinting of the insulin-like growth factor-2 gene in Wilms' tumor. *J Biol Chem* **271**: 9014–9023

Zemel S, Bartolomei M and Tilghman S (1992) Physical linkage of two mammalian imprinted genes, H19 and IGF-2. *Nat Genet* **2**: 61–65

 *© 2000 Cancer Research Campaign*
